# Resonance Frequency Readout Circuit for a 900 MHz SAW Device

**DOI:** 10.3390/s17092131

**Published:** 2017-09-15

**Authors:** Heng Liu, Chun Zhang, Zhaoyang Weng, Yanshu Guo, Zhihua Wang

**Affiliations:** 1 Tsinghua National Laboratory for Information Science and Technology, Institute of Microelectronics, Tsinghua University, Beijing 100084, China; liuhengthu09@outlook.com (H.L.); wengzy13@mails.tsinghua.edu.cn (Z.W.); guoys@tsinghua-sz.org (Y.G.); zhihua@tsinghua.edu.cn (Z.W.); 2 Research Institute of Tsinghua University in Shenzhen, Shenzhen 518057, China

**Keywords:** sensor readout circuit, SAW device, resonance frequency, phase-locked loop, successive approximation

## Abstract

A monolithic resonance frequency readout circuit with high resolution and short measurement time is presented for a 900 MHz RF surface acoustic wave (SAW) sensor. The readout circuit is composed of a fractional-N phase-locked loop (PLL) as the stimulus source to the SAW device and a phase-based resonance frequency detecting circuit using successive approximation (SAR). A new resonance frequency searching strategy has been proposed based on the fact that the SAW device phase-frequency response crosses zero monotonically around the resonance frequency. A dedicated instant phase difference detecting circuit is adopted to facilitate the fast SAR operation for resonance frequency searching. The readout circuit has been implemented in 180 nm CMOS technology with a core area of 3.24 mm^2^. In the experiment, it works with a 900 MHz SAW resonator with a quality factor of Q = 130. Experimental results show that the readout circuit consumes 7 mW power from 1.6 V supply. The frequency resolution is 733 Hz, and the relative accuracy is 0.82 ppm, and it takes 0.48 ms to complete one measurement. Compared to the previous results in the literature, this work has achieved the shortest measurement time with a trade-off between measurement accuracy and measurement time.

## 1. Introduction

The surface acoustic wave (SAW) sensor is a promising multi-functional sensor for pressure [1], mass [2] and temperature [3] measurement in Internet-of-everything (IoE), labs-on-a-chip, biomedical applications [4,5,6,7,8,9]. A SAW device is composed of a piezoelectric substrate and inter-digital transducers (IDT) deposited on the substrate [10]. A resonance frequency readout circuit is required to convert the sensor resonance frequency, which is correlated to the specific physical parameter of the SAW device under measurement, into digital numbers for further processing. Many of the recently published MEMS resonance sensors operate with frequencies in the MHz range [11,12,13,14,15,16], while some others work at a few GHz [17,18]. In this work, the resonance frequency readout circuit has been designed for a 900 MHz one-port SAW device presented in [19] which has a trade-off between the power consumption and the sensitivity consistency. The one-port SAW device in the experiment was fabricated on a LiNbO_3_/SiO_2_/Si substrate based on the ion implantation and the wafer bonding. The first step is to implant He^+^ ions into a LiNbO_3_ wafer. Then a SiO_2_ layer is deposited on a Si wafer. The two prepared wafers are cleaned and bonded together and the bonded pair is heated. Finally, a thin layer of single-crystal LiNbO_3_ is split off from the original LiNbO_3_ wafer and stays on the surface of the SiO_2_/Si structure. A further annealing step is used to increase the bonding strength. Finally, a chemical-mechanical polishing (CMP) process is adopted to smooth the surface of the LiNbO_3_ film. This SAW device has shown improved temperature performance, and more details about the device can be found in [19].

For the readout circuit, the frequency resolution *Δf_min_* and the relative accuracy *Δf_min_/f_o_* (*f_o_* is the nominal resonance frequency) are the key specs of concern. Other key parameters include the power consumption *P* and the time to complete one measurement, which is denoted as *T_MEAS_*. Conventional resonance frequency readout circuits can be classified into two types, the time-based measurement architecture and the frequency-based architecture. Nevertheless, the previous time-based or frequency-based resonance sensor readout circuits either require large *T_MEAS_* [11,12,13,14,15,16] or have a limited relative accuracy [17,18,20,21], inherent from the circuit architectures.

In the time-based measurement architectures [11,12,13,14,15,16], an oscillator or an excitation source, which includes the SAW device in its loop, is counted by a digital counter to determine the resonance frequency. Usually, the time-based method is used for resonators of a few MHz, and cannot be directly used for sensors resonating at around 1 GHz. In [22], the time-based method was used for a 1 GHz resonator in an indirect manner by converting the RF signal down to lower frequency bands. The principle is to count the cycle length of the sensor oscillation signal using a reference clock *f_CLK_*. It takes at least one clock cycle (in terms of the reference clock) to complete one measurement, which means *T_MEAS_* ≥ 1/*f_CLK_*. A higher resolution will always end up in a long measurement time much larger than 1/*f_CLK_*. In general, the main drawback of these architectures lies on the long measurement time *T_MEAS_* if high resolution is needed.

The frequency-based measurement architectures [17,18,20,21] are used to shorten the measurement time with the help of phase-locked loops (PLLs) and successive approximation (SAR) operation [23,24]. The sensor works as the load of the voltage-controlled oscillator (VCO) inside the PLL. In brief, the frequency-based methods show shorter measurement time than the time-based methods, but the frequency resolution is still limited if very small *T_MEAS_* is wanted.

In this paper, a phase-based resonance frequency readout circuit is proposed for the one-port SAW sensors, using a SAR measurement strategy. The presented architecture achieves high accuracy and short measurement time simultaneously with the aid of a fractional-N phase-locked loop (PLL) and a phase difference detecting circuit for resonance frequency searching. The readout circuit is designed in 180 nm CMOS technology. When tested with the 900 MHz SAW device, the readout circuit shows 0.82 ppm relative accuracy while consuming 7 mW power, and it requires 0.48 ms to complete one measurement. The measurement time and accuracy performance makes it distinguished from the reported results in literature.

## 2. Proposed Method

### 2.1. Working Principle

**Figure 1:**
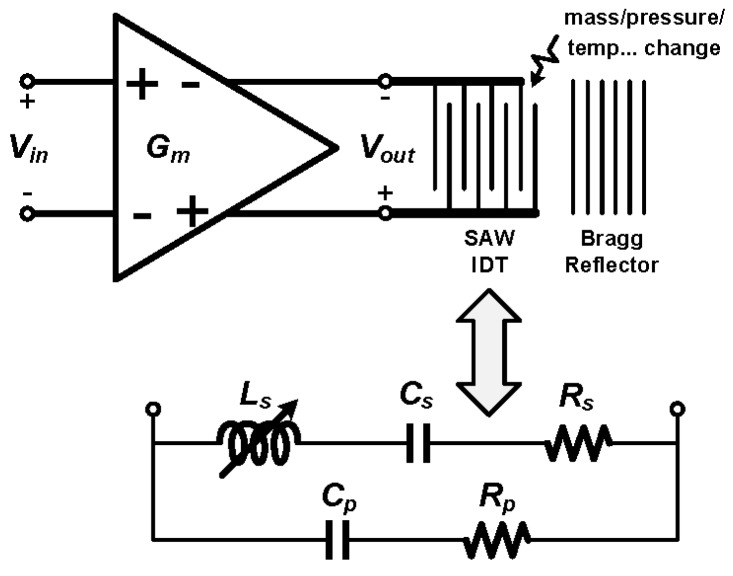
Equivalent circuit model of a one-port SAW device (*G_m_* represents the input driver of the SAW IDT, the RLC model of the SAW IDT is shown in the lower part).

**Figure 2:**
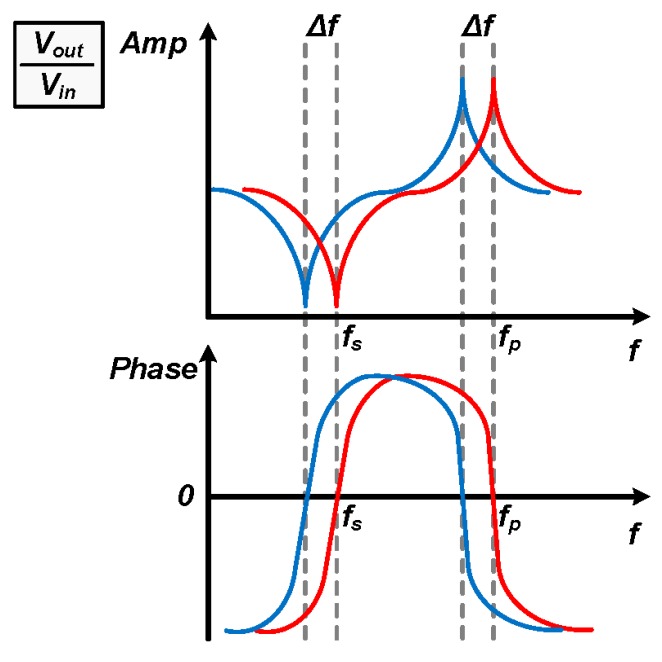
Frequency response of a driving amplifier when loaded by a one-port SAW device (upper part: amplitude-frequency response, lower part: phase-frequency response).

A one-port SAW device together with a driving amplifier is shown in the upper part of Figure 1. The equivalent RLC circuit model [25] is shown in the lower part of Figure 1. The one-port SAW device has two resonance points, the parallel one *f_p_* and the series one *f_s_* [25,26], which can be observed in the frequency response of the driving amplifier’s input-output transfer characteristic when loaded by the SAW device. Both the parallel and series resonance shown in Figure 2, frequencies may have a small shift *Δf* correlated to the change in pressure, loaded mass, or temperature applied to the SAW device. The one-port SAW device can serve as a pressure/mass/temperature sensor if the resonance frequency can be read out precisely [19]. The frequency shift *Δf* is proportional to the change in a certain physical parameter *x* (i.e., pressure, loaded mass, or temperature) if the change is sufficiently small.

With a SAW resonator as the load, the driving amplifier shows the parallel resonance at the frequency *f_p_* and the series resonance at the frequency *f_s_*. As shown in Figure 2, the driving amplifier’s amplitude-frequency response has a peak around *f_p_* and a notch around *f_s_*. With a measurement circuit with limited voltage resolution, it is desired to choose the parallel resonance for the magnitude measurement. Hence the readout circuit in this work is designed to measure the parallel resonance frequency, and the “resonance frequency” *f_0_* in the remaining part of this paper refers to the parallel resonance frequency *f_p_*.

**Figure 3:**
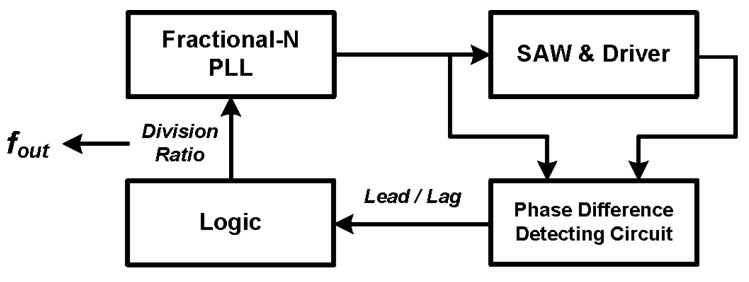
Block diagram of the proposed architecture, including the SAW under test and its driver circuit, the fractional-N PLL as test stimulus, the phase difference detecting circuit and the control logic for working flow control.

**Figure 4:**
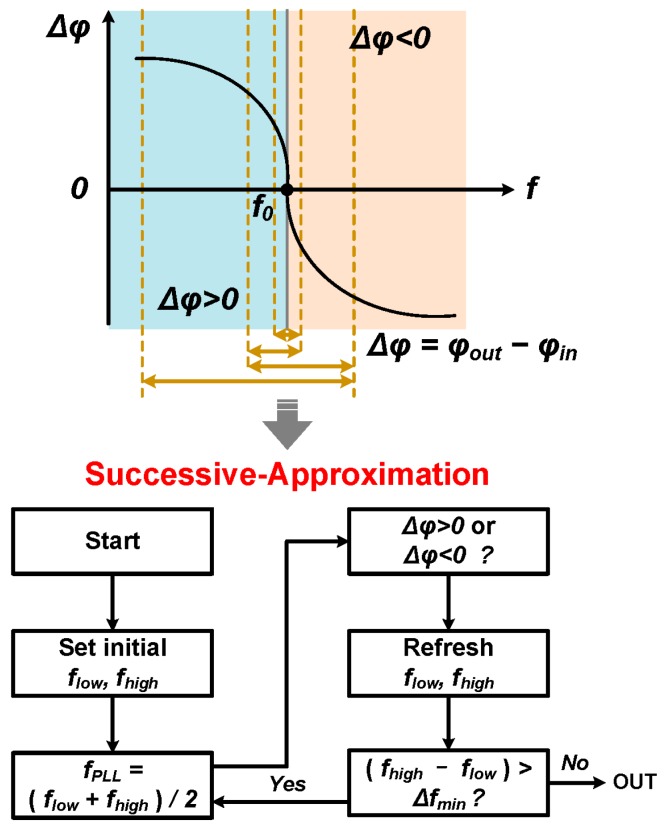
The basic principle of the proposed architecture (upper part: the resonance frequency *f_0_* corresponds to *Δφ* close to 0 in the phase-frequency response, the lower part: the flow chart to find the resonance frequency *f_0_*).

In this work, a phase-based new architecture is proposed to optimize the measurement time and frequency resolution at the same time, and its block diagram is shown in Figure 3. A fractional-N PLL with high frequency resolution is employed as the frequency generator. The sensor serves as the load of the output driving circuit (driver). The base of this method is the phase difference *Δφ* between the driver input and output signals, which is zero around the SAW device resonance frequency *f_0_*, as illustrated in Figure 4.

The phase curve in Figure 4 is actually an enlarged view of Figure 2 around the parallel resonance frequency *f_p_*. *Δφ* is the phase difference between the input and output of the SAW driver. The phase difference is monotonic and crosses zero right at the resonance frequency. The task of the resonance frequency readout is then to find the frequency that gives *Δφ* = 0, and this is where the SAR algorithm comes in. The flow chart of the SAR algorithm is shown in the lower part of Figure 4. The frequency searching starts from an initial frequency range [*f_low_*, *f_high_*], and the PLL/SAW driver output frequency is set to (*f_low_* + *f_high_*)/2 at beginning. If this frequency gives *Δφ* < 0, then the next frequency range will be [*f_low_*, (*f_low_* + *f_high_*)/2]. Otherwise, the next frequency range will be [(*f_low_* + *f_high_*)/2, *f_high_*]. This operation is repeated until the frequency searching range is less than the wanted frequency resolution *Δf_min_.* As an example, Figure 4 shows how a three-step search goes. The idea of phase difference measurement for sensor readout can also be found in [27]. The key contribution of this work is that the phase difference is directly measured using the circuits presented in this paper, while in [27] the phase difference was measured using an indirect time-based method, which requires more measurement time.

### 2.2. Circuit Architecture

**Figure 5:**
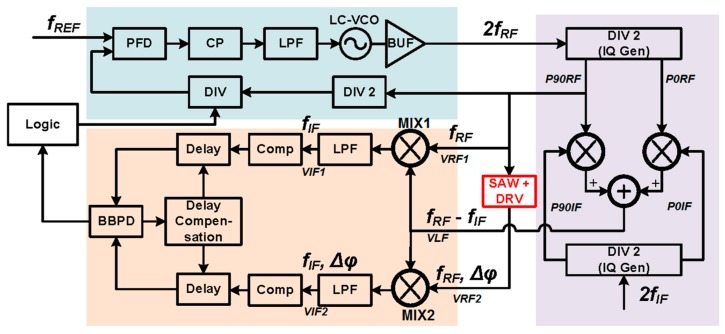
Detailed block diagram of the proposed readout circuit, including the SAW and its driver in the center, the PLL in the upper-left part, the quadrature signal generator in the right, and the phase difference detecting circuit in the lower-left part).

The detailed block diagram of the readout circuit is shown in Figure 5. The left down part is the phase difference detecting circuit. A fractional-N PLL is used to generate the RF excitation. Both the input and output signals of the SAW driver are around GHz range. There are three critical parts in the readout circuit. The first one is the binary phase difference detecting circuit, which judges the sign of *Δφ*. The second one is the logic circuit, which controls the PLL output frequency to search for the resonance frequency with the SAR strategy. The third one is the fractional-N PLL as a stimulus source. The SAR operation starts between two frequencies *f_high_* and *f_low_*, which can guarantee to cover the resonance frequency, and in this design *f_high_* and *f_low_* are set to 816 MHz and 1008 MHz, respectively. Since the PLL fractional divider has finite resolution, the searching will stop if the frequency searching range reaches the least significant bit (LSB) step size of the PLL output frequency. The readout circuit frequency resolution is actually mostly determined by the PLL frequency setting resolution.

The VCO output frequency in the PLL is two times the SAW device resonance frequency, such that the quadrature signals can be easily generated. In Figure 5, the input frequency to the SAW device is denoted as *f_RF_*, and the PLL output frequency is then 2*f_RF_*. The PLL output frequency is divided by 2 to generate the quadrature signals *P90_RF_* = cos (2*πf_RF_t*) and *P0_RF_* = sin (2*πf_RF_t*). *P90_RF_* is sent to the SAW driver loaded by the SAW device (the “SAW + DRV” block in Figure 5). Compared to its input signal, the driver output has a phase difference denoted as *Δφ*. The input signal and the output signal of the driver can be written as

(1) $$\left\{ \begin{array}{l} V_{RF1}=A_{RF}\cos\left(2\pi f_{RF}t\right)\\ V_{RF2}=A_{RF}\cos\left(2\pi f_{RF}t+\Delta \varphi \right) \end{array}\right.$$

If roughly assume *f_RF_* = 1 GHz and *Δφ* = 1°, the time difference between *V_RF_*_1_ and *V_RF_*_2_ is only 2.78 ps. It is definitely non-trivial to measure this tiny time difference. Frequency division is of no use here because the phase difference *Δφ* is also divided which is not desired. In this work, the down-mixers MIX1 and MIX2 are adopted to hold the value of *Δφ* while greatly reducing the input signal frequency of the phase difference detecting circuit. Both *V_RF_*_1_ and *V_RF_*_2_ are down-converted to the intermediate frequency (IF). If the desired IF frequency is *f_IF_*, another frequency signal *V_LF_* with a frequency equal to *f_RF_*-*f_IF_* is generated and sent to MIX1 and MIX2.

(2) $$V_{LF}=A_{LF}\cos\left(2\pi (f_{RF}-f_{IF})t\right)$$

The output signals of MIX1 and MIX2 are given as follows

(3) $$\left\{ \begin{array}{cc} V_{RF1}\cdot V_{LF} & =\frac{A_{RF}A_{LF}}{2}\left(\cos\left(2\pi f_{IF}t\right)+\cos\left(2\pi \left(2f_{{}_{RF}}-f_{IF}\right)t\right)\right)\\ V_{RF2}\cdot V_{LF} & =\frac{A_{RF}A_{LF}}{2}\left(\cos\left(2\pi f_{IF}t+\Delta \varphi \right)+\cos\left(2\pi \left(2f_{{}_{RF}}-f_{IF}\right)t+\Delta \varphi \right)\right) \end{array}\right.$$

Both mixers’ output signals pass through a low-pass filter (LPF), and the generated IF signals *V_IF_*_1_ and *V_IF_*_2_ are given as

(4) $$\left\{ \begin{array}{l} V_{IF1}=A_{IF}\cos(2\pi f_{IF}t)\\ V_{IF2}=A_{IF}\cos(2\pi f_{IF}t+\Delta \varphi ) \end{array}\right.$$

It is clearly seen that the phase difference *Δφ* between *V_RF_*_1_ and *V_RF_*_2_ is converted to the same phase difference between the IF signals *V_IF_*_1_ and *V_IF_*_2_. Again take *Δφ* = 1° as a numerical example. If the IF frequency *f_IF_* is 200 kHz, after down-conversion, the time difference between *V_IF_*_1_ and *V_IF_*_2_ is now 13.9 ns, and this time difference can be easily measured. In this design, the IF signal time difference is detected by a digital bang-bang phase detector (BBPD) afterwards.

The signal *V_LF_* with frequency *f_RF_*-*f_IF_* is generated using a quadrature mixer as shown in the right part of Figure 5. The IF quadrature divider first generate the quadrature signals *P90_IF_* = cos (2*πf_IF_t*) and *P0_IF_* = sin (2*πf_IF_t*). In this design, *f_IF_* = 200 kHz. *V_LF_* is then generated by mixing *P90_RF_*/*P0_RF_* and *P90_IF_*/*P0_IF_* as follows.

(5) $$V_{LF}=A_{RF}A_{IF}\left(\cos\left(2\pi f_{RF}t\right)\cos\left(2\pi f_{IF}t\right)+\sin\left(2\pi f_{RF}t\right)\sin\left(2\pi f_{IF}t\right)\right)=A_{RF}A_{IF}\cos\left(2\pi \left(f_{RF}-f_{IF}\right)t\right)$$

### 2.3. Performance Analysis

If a *B*-bit fractional divider (DIV) is used in the PLL in Figure 5, the frequency resolution of the *f_RF_* output is

(6) $$\Delta f_{\min}=\frac{2f_{REF}}{2^{B}}$$

The factor 2 in the numerator is due to the extra divide-by-2 divider DIV2 between the fractional divider DIV and the SAW drive. It is desired to slow down the IF signals signal to make the time difference large enough for measurement, but it will increase the measurement time inevitably. Hence it is important to find the lowest IF frequency allowed. Take the BBPD into consideration and the IF signal cycle period *T_IF_* is constrained by the minimum phase shift.

(7) $$\frac{\Delta t_{PD}}{T_{IF}}\leq \frac{\Delta \varphi_{\min}}{2\pi }$$

in which *Δt_PD_* represents the minimum time difference that the BBPD can tell correctly, in other word, the deadzone. The minimum phase difference can be derived as

(8) $$\Delta \varphi_{\min}=\left|{\frac{\partial \varphi }{\partial f}|}_{f=f_{0}}\right|\cdot \Delta f_{\min}$$

in which *φ*(*f*) is a function of frequency which describes the phase-frequency response of the SAW device as shown in Figure 4. To find the value of the partial derivative, an equivalent parallel RLC resonance circuit is used to analogy the sensor around the resonance frequency. The phase difference is given by

(9) $$\varphi =-\arctan\left(R\left(\frac{1}{2\pi fL}-2\pi fC\right)\right)$$

The partial derivative around *f_0_* can be expressed by the Q factor as

(10) $${\frac{\partial \varphi }{\partial f}|}_{f=f_{0}}=-\frac{2Q}{f_{0}}\;(Q=\frac{R}{2\pi f_{0}L}=2\pi f_{0}CR)$$

Combine (6)–(8) and (10), and the minimum IF signal cycle period limited by the BBPD is

(11) $$T_{IF,\min}=\frac{\pi f_{0}\Delta t_{PD}}{2f_{REF}Q}\cdot 2^{B}$$

For a *B*-bit fractional divider, it takes *B* searching steps to get the final result using SAR. Consequently, *T_MEAS_* is limited by *B•T_IF,min_*, which can written as

(12) $$T_{MEAS,BBPD}=B\frac{\pi f_{0}\Delta t_{PD}}{2f_{REF}Q}\cdot 2^{B}$$

Another limiting factor on *T_MEAS_* is the PLL settling time. *T_MEAS_* should be no shorter than the PLL settling time times *B*. The PLL settling time is limited by the PLL bandwidth *BW_PLL_*, and the bandwidth is usually a fraction of its reference frequency *f_REF_*. Here we assume that time of each searching step is *α* times the reference clock cycle period 1/*f_REF_*. *T_MEAS_* limited by the PLL is given by

(13) $$T_{MEAS,PLL}\propto B\cdot \frac{1}{BW_{PLL}}\propto B\cdot \frac{1}{f_{REF}},\;T_{MEAS,PLL}=B\cdot \frac{\alpha }{f_{REF}}$$

For a regular PLL design, *f_REF_* is about tens times *BW_PLL_* [28], and therefore *α* will not exceed 100.

In order to satisfy the restrictions of both the BBPD and the PLL settling time, the minimum measurement time is givens as

(14) $$T_{MEAS,\min}=\max\left\{B\frac{\pi f_{0}\Delta t_{PD}}{2f_{REF}Q}\cdot 2^{B},\frac{B\alpha }{f_{REF}}\right\}$$

To find the optimal design parameters, a measurement figure of merit *FoM* is defined to relate the measurement time *T_MEAS_* and the frequency measurement resolution *Δf_min_*. Obviously, it is wanted to have a small *FoM.*

(15) $$FoM=T_{MEAS}\Delta f_{\min}$$

With (6) and (15), we have

(16) $$FoM=\max\left\{\frac{B\pi f_{0}\Delta t_{PD}}{Q},\frac{2\alpha B}{2^{B}}\right\}$$

**Figure 6:**
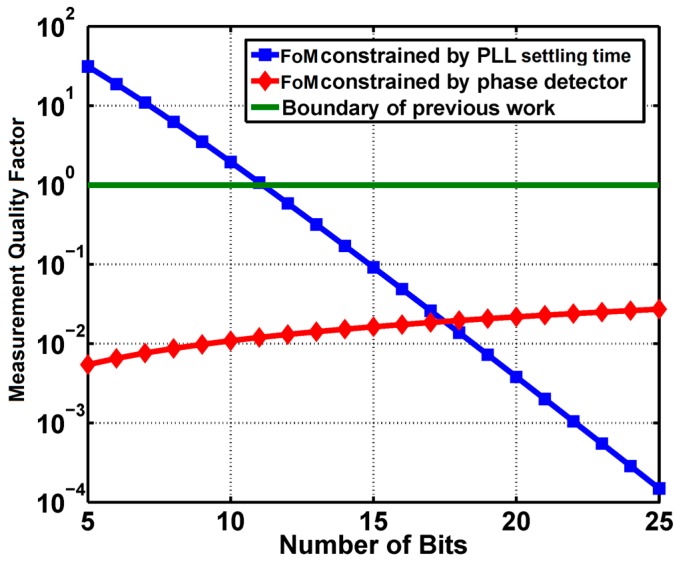
Measurement figure of merit *FoM* vs. bits number of PLL fractional divider, including FoM constrained by PLL and phase detector, and the boundary of previous work for comparison.

To show *Q_M_* of the proposed architecture quantitatively, some numbers from the real circuit with the proposed circuit are used. The reference frequency *f_REF_* is 24 MHz, the SAW resonance frequency is about 900 MHz, and its Q factor is 130. *Δt_PD_* is obtained from the worst case (SS corner, 80% power supply, 85 °C) simulation, which is 50 ps. With these values in hand, we can plot the measurement quality factor *FoM* constrained by the PLL settling time and the phase detector versus the bits number of the PLL fractional divider, as shown in Figure 6. The *FoM* limit of the previous work [11,12,13,14,15,16,17,18] is also shown in Figure 6.

As shown in Figure 6, the intersection of the *FoM* curve limited by the PLL and that limited by the phase detector suggests an optimal divider bits number. The optimal point can also be found by solving $\frac{B\pi f_{0}\Delta t_{PD}}{Q}=\frac{2\alpha B}{2^{B}}$, which gives

(17) $$B_{opt}=\log_{2}\frac{2Q\alpha }{\pi f_{0}\Delta t_{PD}}$$

(18) $$FoM_{opt}=\frac{\pi f_{0}\Delta t_{PD}}{Q}\log_{2}\frac{2Q\alpha }{\pi f_{0}\Delta t_{PD}}$$

The optimal *FoM* is determined by *f_0_*, *Q*, *Δt_PD_* and *α*, among which the only circuit design parameter is *α*. For α = 100, which is the upper limit from the previous discussion, *B_opt_* is 18, and the best *FoM* of the proposed architecture is only 0.02. As a contrast, the previous work [11,12,13,14,15,16,17,18] has a *FoM* limit of 1. To sum up, the proposed architecture can achieve a trade-off between the measurement time and frequency resolution, by shrinking their product smaller than that of the previous work.

## 3. Circuit Implementation

The proposed architecture as shown in Figure 5 has been designed and fabricated in a 180 nm CMOS technology. The circuit implementation details and the key design considerations will be given in this section.

### 3.1. Connection Parasitics between the SAW Device

**Figure 7:**
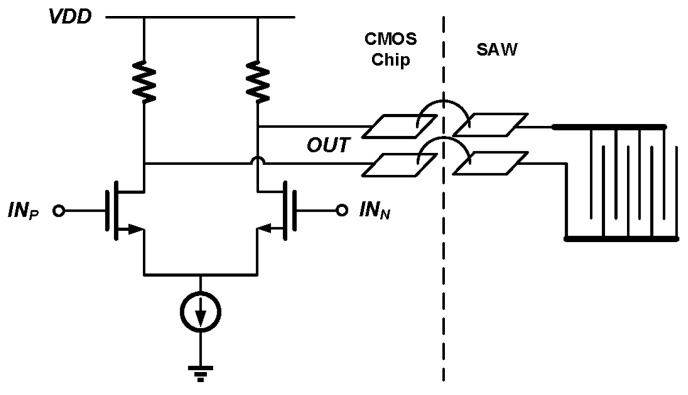
SAW device connected to the driving amplifier in the readout chip using bonding wires.

To implement a compact sensor, the reported resonance frequency readout circuit will be connected to the SAW device using bonding wires, as shown in Figure 7. The SAW driving circuit in the readout chip is a simple differential amplifier with its output nodes connected to two pads. Two bonding wires tie the pads on the CMOS chip and the IDT on the SAW chip together.

The direct measurement on the SAW device shows that its equivalent parallel RLC model has the parallel capacitance *Cp*, parallel inductance *Lp*, and parallel resistance *R_p_* equal to 6.32 pF, 43.1 nH and 254 Ω, respectively, and the *Q* value without the parasitics reaches 163.

**Figure 8:**
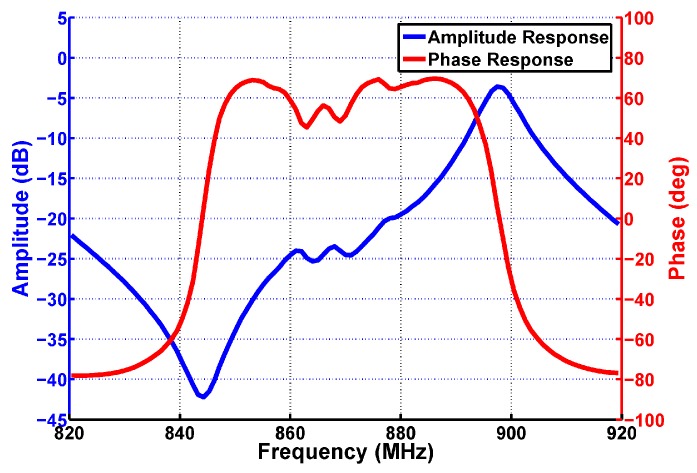
Simulated SAW driving amplifier gain using the measured SAW device S-parameter, including the amplitude-frequency response and the phase-frequency response.

To build the behavior model for the SAW device, the SAW device is directly bonded on the printed circuit board (PCB) and connected to a network analyzer for port characteristic measurement. Then the S-parameter (*S_11_*) file obtained in this way is included in the simulation testbench of the driving circuit, and the result is shown in Figure 8. The parallel resonance frequency is about 898 MHz. The slope at the resonance frequency is 16.5 µdeg/Hz approximately, and the *Q* factor is about 130, according to Equation (10). Note that the parasitic effect has already been taken into consideration in this *Q* value.

It has been confirmed through simulation that the parasitics effects of the bonding pads and wires can be safely ignored.

(1) The driving amplifier output capacitance and the bonding pad parasitic capacitance are actually relatively small compared to the SAW device parallel capacitance *Cp*, and they cause the resonance frequency to shift about −20 kHz. This frequency shift is almost constant in the effective measurement range. It can be calibrated out without affecting the linearity of the resonance frequency detection.

(2) The parasitic resistance causes the qualify factor *Q* to drop from about 163 to 130. This decrease in *Q* may have some effect in the measurement time according to Equation (11). Again, this effect can be easily compensated for by slightly increasing the PLL reference frequency *f_REF_*, if needed.

### 3.2. Fractional-N PLL

**Figure 9:**
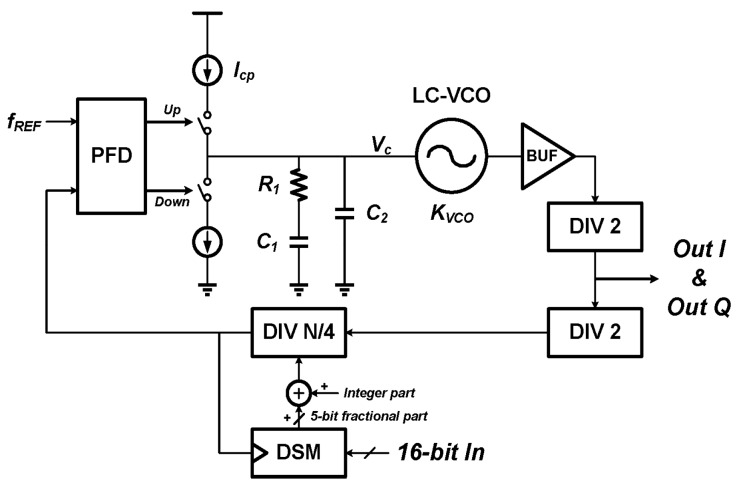
Block diagram of the fractional-N PLL, including the PFD, LPF, VCO and divider.

The block diagram of the fractional-N PLL is shown in Figure 9. A type II third-order charge pump PLL [29,30] with the LC-VCO centered at 1800 MHz is employed in the proposed readout circuit. The reference frequency is chosen to be 24 MHz, and the division ratio of the fractional divider DIV is between 18 and 19. The total division ratio is between 72 and 76, which means that RF signals *V_RF1_* and *V_RF2_* have a frequency that ranges from 864 to 912 MHz, which is actually the measurement range of the readout circuit. A single loop, third order delta sigma modulator (DSM) is used to get the fractional division ratio. It has 16-bit input (*B* = 16, which is close to the *B_opt_* given by (17)) and 5-bit signed output, and the frequency resolution is about 732.42 Hz according to (6). Transient simulations are performed to obtain the measurement time of each searching step limited by the PLL settling time, and it shows that 40 µs turns to be a very safe value with a simulated PLL settling time of ~12.5 µs.

### 3.3. Mixer and I/Q Generator

**Figure 10:**
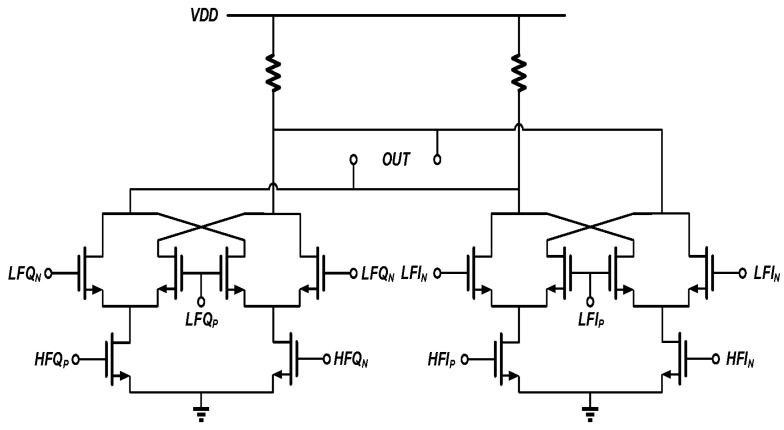
Quadrature mixer to generate *V_L**F**_*. The inputs signal *LF* and *HF* are both quadrature differential, and the output is differential.

There are two kinds of mixers used in the proposed circuit. The first one is the quadrature mixer [31], which provides the *V_LF_* signal as shown in Figure 5. Its circuit is shown in Figure 10. The annotation “HF” in Figure 10 represents the *P90_RF_* and *P0_RF_* as shown in Figure 5, while “LF” stands for the *P90_IF_* and *P0_IF_* signals.

**Figure 11:**
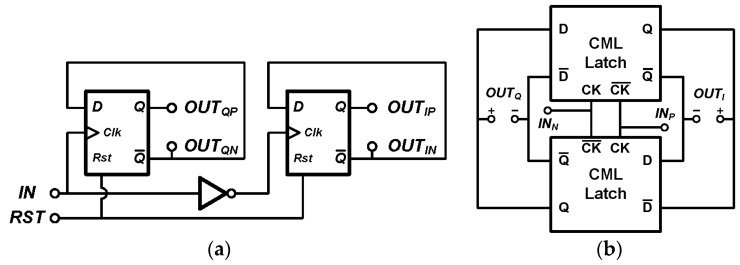
Divide-by-2 dividers with quadrature output (**a**) for IF signal generation; (**b**) for RF signal generation.

*P90_IF_* and *P0_IF_* have a frequency of 200 kHz in this design, and they are generated using the digital circuit as shown in Figure 11a. Hence these two signals have square waveforms and can be expanded in Fourier series as

(19) $$\begin{array}{l} P90_{IF}=\frac{2A_{IF}}{\pi }\sum_{n=0}^{+\infty }\frac{\cos\left(\left(2n+1\right)2\pi f_{IF}t\right)}{{\left(-1\right)}^{n}\left(2n+1\right)}\\ P0_{IF}=\frac{2A_{IF}}{\pi }\sum_{n=0}^{+\infty }\frac{\sin\left(\left(2n+1\right)2\pi f_{IF}t\right)}{2n+1} \end{array}$$

For RF signals *P90_RF_* and *P0_RF_*, two current mode logic (CML) latches are used as shown in Figure 11b. *P90_RF_* and *P0_RF_* mix up with *P90_IF_* and *P0_IF_* according to (5). The output of the quadrature mixer is

(20) $$V_{LF}=\frac{2A_{IF}A_{RF}}{\pi }\sum_{n=0}^{+\infty }\frac{\cos2\pi \left(f_{RF}-{\left(-1\right)}^{n}(2n+1)f_{IF}\right)t}{{\left(-1\right)}^{n}{\left(2n+1\right)}^{2}}$$

In this design, *f_RF_* and *f_IF_* are chosen to be 900 MHz and 200 kHz, respectively. Equation (20) shows that the actual *V_LF_* is not a single tone signal.

**Figure 12:**
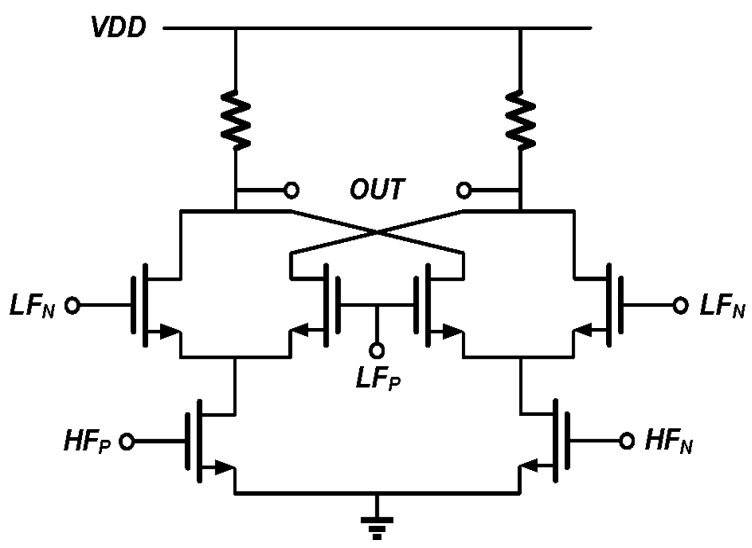
Circuit of the down-mixer. The inputs *HF* and *LF* are differential, and so is the output.

The second kind of mixer is the down-mixer MIX1 and MIX2 that convert the phase difference to the IF band. The circuit is shown in Figure 12 where the “HF” refers to *V_RF1_* and *V_RF2_*, which are the input/output of the SAW device driving amplifier, and “LF” refers to the quadrature mixer output *V_LF_*. *V_IF1_* and *V_IF2_* from Equations (3) and (4) need to be re-checked since *V_LF_* is no longer a single tone. The actual *V_IF1_* and *V_IF2_* can be written as

(21) $$\left\{ \begin{array}{l} V_{IF1}=\frac{2A_{IF}A_{RF}^{2}}{\pi }\sum_{n=0}^{+\infty }\frac{\cos\left(2\pi {\left(-1\right)}^{n}(2n+1)f_{IF}t\right)}{{\left(-1\right)}^{n}{\left(2n+1\right)}^{2}}\\ V_{IF2}=\frac{2A_{IF}A_{RF}^{2}}{\pi }\sum_{n=0}^{+\infty }\frac{\cos\left(2\pi {\left(-1\right)}^{n}(2n+1)f_{IF}t+\Delta \varphi \right)}{{\left(-1\right)}^{n}{\left(2n+1\right)}^{2}} \end{array}\right.$$

It can be shown that the harmonics have no effect on the lead-lag relationship between *V_IF1_* and *V_IF2_.* This can be done by checking the zero-crossing points of *V_IF1_* and *V_IF2_*. It is obvious that *t_zc_*(*k*) = (2*k* + 1)/4*f_IF_* (*k* = 0, 1, 2, …) are the zero-crossing points of *V_IF1_*. A quick numeric simulation using Matlab shows that the harmonics will not create any extra zero-crossing point other than *t_zc_*(*k*).

For any *k*, *V_IF1_*(*t_zc_*(*k*)) = 0,$\frac{\cos\left(2\pi {\left(-1\right)}^{n}(2n+1)f_{IF}t_{zc}(k)\right)}{{\left(-1\right)}^{n}{\left(2n+1\right)}^{2}}=0$, the slope of *V_IF1_* at the time point *t_zc_*(*k*) is

(22) $${\frac{dV_{IF1}}{dt}|}_{t=t_{zc}(k)}=A_{IF}A_{RF}^{2}\sum_{n=0}^{+\infty }\frac{(2k+1)\sin\left(\frac{\pi }{2}{\left(-1\right)}^{n}(2n+1)(2k+1)\right)}{\left(2n+1\right)}$$

First check the case that *k* is odd. When *k* is odd, at the time point *t_zc_*(*k*),

(23) $${\frac{dV_{IF1}}{dt}|}_{t=t_{zc}(k)}=-(2k+1)A_{IF}A_{RF}^{2}\sum_{n=0}^{+\infty }\frac{\sin\left(\frac{\pi }{2}{\left(-1\right)}^{n}(2n-1)\right)}{\left(2n+1\right)}\;=-(2k+1)A_{IF}A_{RF}^{2}\left(-1-\frac{1}{3}-\frac{1}{5}-\ldots \right)>0$$

(24) $$\begin{array}{cc} V_{IF2}(t_{zc}(k)) & =-\frac{2A_{IF}A_{RF}^{2}}{\pi }\sum_{n=0}^{+\infty }\frac{\sin\left(2\pi {\left(-1\right)}^{n}(2n+1)f_{IF}t_{zc}\right)\sin\Delta \varphi }{{\left(-1\right)}^{n}{\left(2n+1\right)}^{2}}\\ & =\left(-\frac{2A_{IF}A_{RF}^{2}}{\pi }\left(-1+\frac{1}{3^{2}}-\frac{1}{5^{2}}+\frac{1}{7^{2}}-\ldots \right)\right)\sin\Delta \varphi \end{array}$$

(24) shows that *V_IF2_*(*t_zc_*(*k*)) has the same polarity as sin(Δ*φ*).

If *V_RF1_* is slightly leading *V_RF2_*, we have –π < Δ*φ* < *0* and sin (Δ*φ*) < 0. (23) and (24) shows that if *k* is odd, when *V_IF1_* crosses zero with a positive slope, *V_IF2_* is still below zero. Under this case *V_IF1_* is still leading *V_IF2_*, just as *V_RF1_* is leading *V_RF2_*.

The same conclusion can be made when *k* is even. This analysis has validated that harmonics in *V_LF_* have no effect on the lead-lag relationship between *V_IF1_* and *V_IF2_*.

On the other hand, the distortion caused by the CML nonlinearity has been checked using the transistor level simulation, and it is also proven that the CML nonlinearity can also be neglected.

### 3.4. Passive LPF and Comparator

In this design, the low past filter (LPF) is placed after the down-mixer to filter out the high frequency components, and the *V_IF1_* and *V_IF2_* signal can only contain the sub-1 MHz components for phase difference detection.

**Figure 13:**
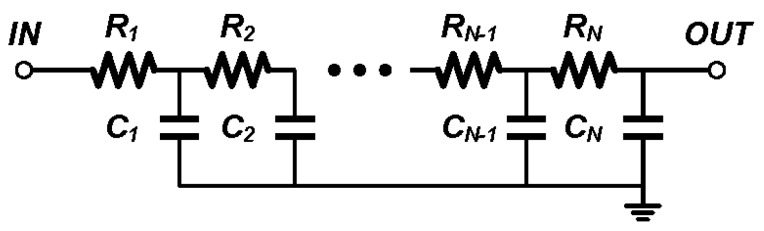
Passive RC LPF to remove the high frequency component in *IF* signals.

A passive RC filter is used to save power consumption. A simple realization is shown in Figure 13, where all the resistors and capacitors are chosen to be identical for simplicity. The third order passive LPF is chosen as a trade-off between stop-band attenuation and area.

**Figure 14:**
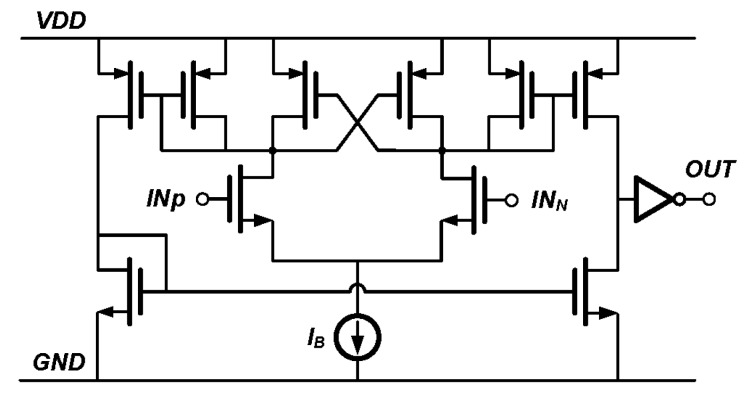
Schematic of the comparator, with cross coupled transistors as load for fast comparison.

The comparator (“Comp” in Figure 5) circuit is shown in Figure 14. The comparator is a open-loop amplifier with negative resistance transistors to increase the gain bandwidth [32]. In this readout chip, the comparator actually serves as a differential to a single-ended converter that converts the differential analog input *V_IF1_* and *V_IF2_* into digital pulses.

### 3.5. BBPD and Control Logic

**Figure 15:**
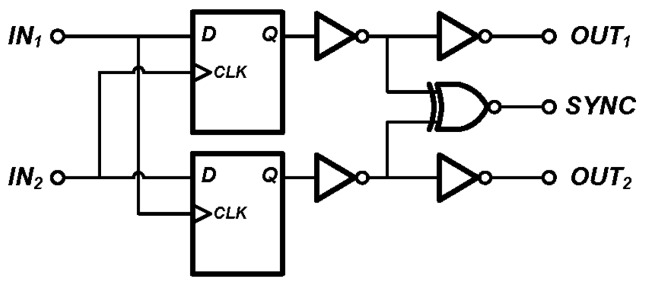
Block diagram of the BBPD, used to detect the phase difference of 2 input signals.

The circuit of the BBPD is given in Figure 15. True single phase clock (TSPC) registers [33] are used here. The output “*OUT*_1_” and “*OUT*_2_” indicate whether “*IN*_1_” is leading “*IN*_2_” or vice versa. The output “*SYNC*” will be high if the time difference between the input signals is too small for the BBPD to distinguish, in other words, the input signals are “synchronous”.

The SAR searching control logic is composed of 3 parts, the delay compensation, the VCO capacitor bank preset and the SAR algorithm. The first and second parts are used only before the real measurements start. The delay compensation block is used to compensate for the delay mismatch between the two signal paths, i.e., the path of *V_RF1_*/*V_IF1_* and the path of *V_RF2_*/*V_IF2_*. Two digital controlled delay lines as shown in Figure 5 are tuned to cancel the delay mismatch using on a logic control circuit with the BBPD “*SYNC*” as its input.

The IF frequency is chosen to be 200 kHz, meeting the requirement of (11). The initial searching step time *T_STEP_* is 40 µs, which equals to 8 cycles of the IF signal (200 kHz). However, the frequency step becomes smaller as the binary search goes, and a variable searching step time *T_STEP_* is used in this design to shorten the overall measurement time *T_MEAS_*. *T_STEP_* is set to 8 IF signal cycles to determine 4 MSB bits of the PLL frequency setting, 4 IF cycles for the 4 LSB bits and 6 IF cycles for the 8 intermediate bits. Thus, it takes 96 IF signal cycles, which is 0.48 ms, to complete one measurement.

## 4. Experimental Results

### 4.1. Measurement Set-up

**Figure 16:**
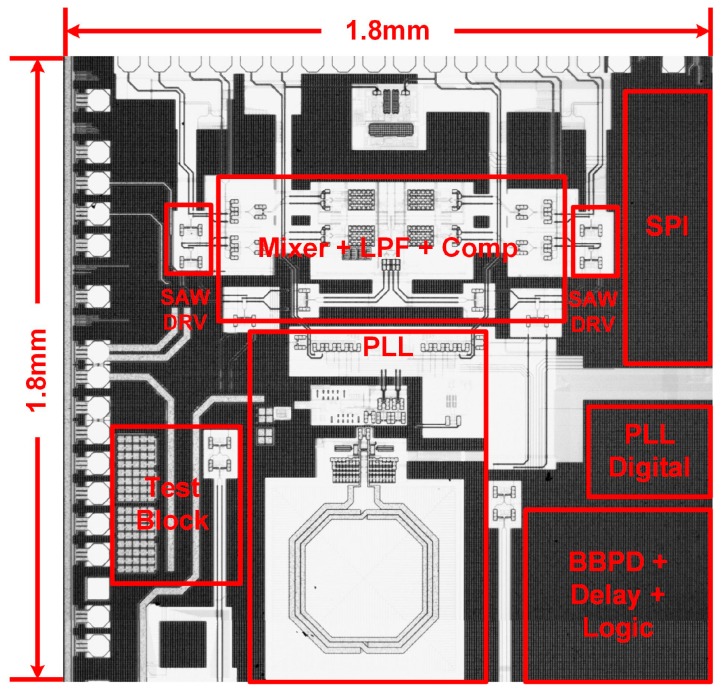
Readout circuit chip micrograph.

**Figure 17:**
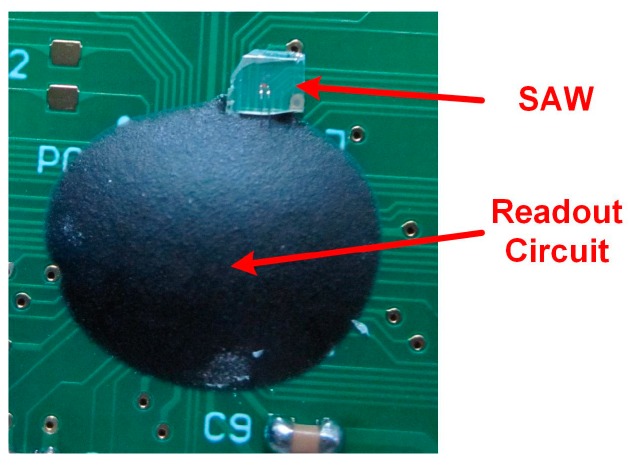
Chip-on-board (CoB) package of the readout chip and SAW device.

The proposed readout circuit was implemented and fabricated in a 180 nm CMOS technology. The chip micrograph is shown in Figure 16. The core area of the circuit is about 1.8 mm × 1.8 mm. The readout circuit chip and the SAW device are connected together via bonding wires according, as shown in Figure 17.

**Figure 18:**
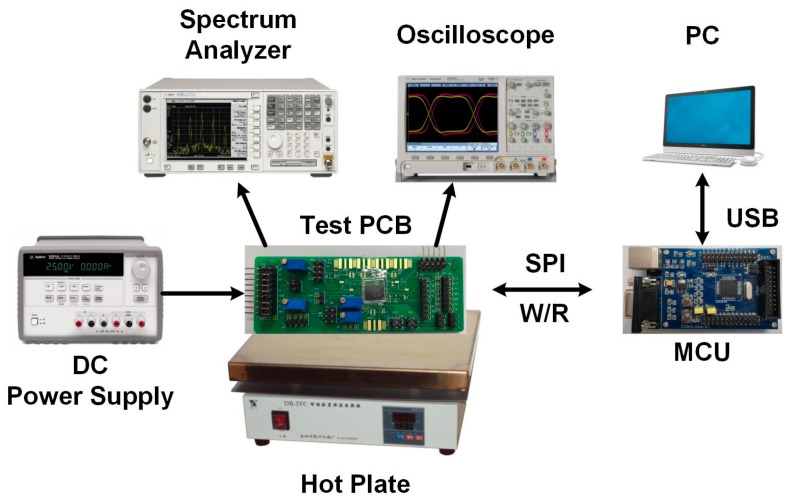
Measurement set-up. The proposed chip and the SAW device are on the test PCB, and the test PCB is controlled by a MCU board which is further connected to PC for data collection.

The measurement set-up in which the SAW device and the resonance frequency readout chip are used as a temperature sensor is shown in Figure 18. The test PCB is placed on a hot plate. An external 24 MHz clock signal is used as the PLL reference clock. A microcontroller (MCU) is employed to read/write the control words via a serial peripheral interface (SPI). Firstly, the temperature of hot plate is set to 25 °C. A spectrum analyzer records the PLL output spectrum. An oscilloscope is used to evaluate the PLL settling time and the phase difference detecting circuit. Secondly, the temperature of hot plate is set from 25 to 55 °C with a 5 °C step, by which the function of the proposed readout circuit is verified.

### 4.2. Experimental Results

**Figure 19:**
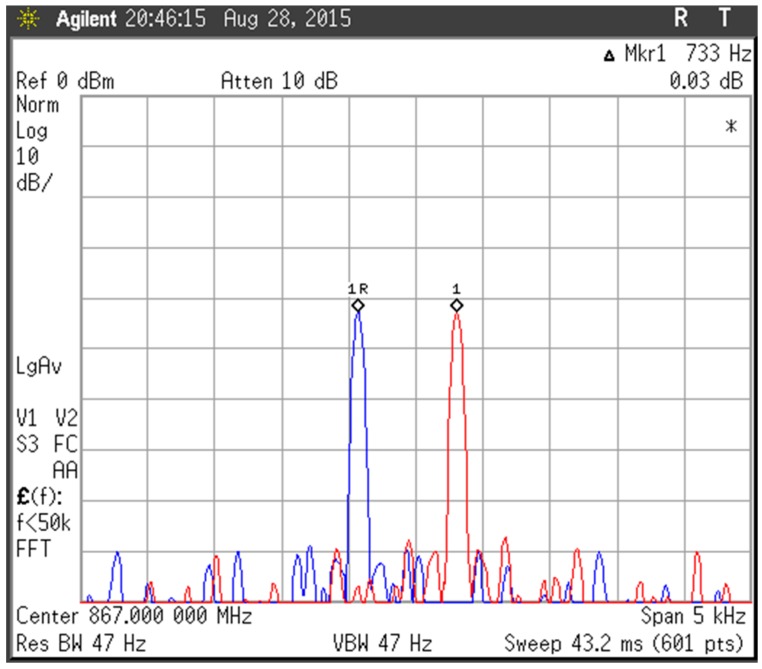
Measured frequency resolution of the fractional-N PLL. The two curves are the spectra of PLL’s two outputs with 733 Hz frequency difference which is controlled by configuring the PLL frequency setting register.

The resonance frequency resolution is measured first. By setting the frequency the PLL fractional division ratio with a difference of 2^−16^, the chip gave the RF signals with the minimum frequency difference, which is actually the resonance frequency resolution. The spectra of the two signals is shown in Figure 19, and the frequency resolution is 733 Hz, which agrees with the value of 732.42 Hz predicted by (6). The relative resolution is about 0.82 ppm with respect to the nominal measurement frequency of 900 MHz.

**Figure 20:**
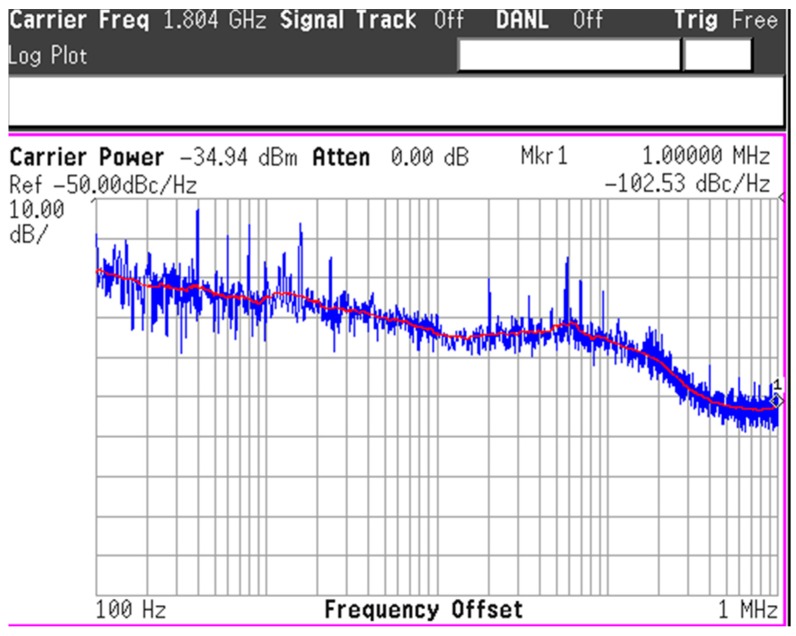
Measured phase noise of the fractional-*N* PLL.

Figure 20 gives the measured phase noise of the VCO output centered at about 1.8 GHz when the PLL loop is locked. The phase noise is −95.29 dBc/Hz at 100 kHz frequency offset and is −102.53 dBc/Hz at 1 MHz frequency offset. It should be emphasized that it has been verified through behavior simulation that such noise level will not affect the frequency measurement resolution. The key non-idealities that affect the measurement accuracy with limited measurement time are the PLL settling time and the minimum phase difference that the BBPD can differentiate.

**Figure 21:**
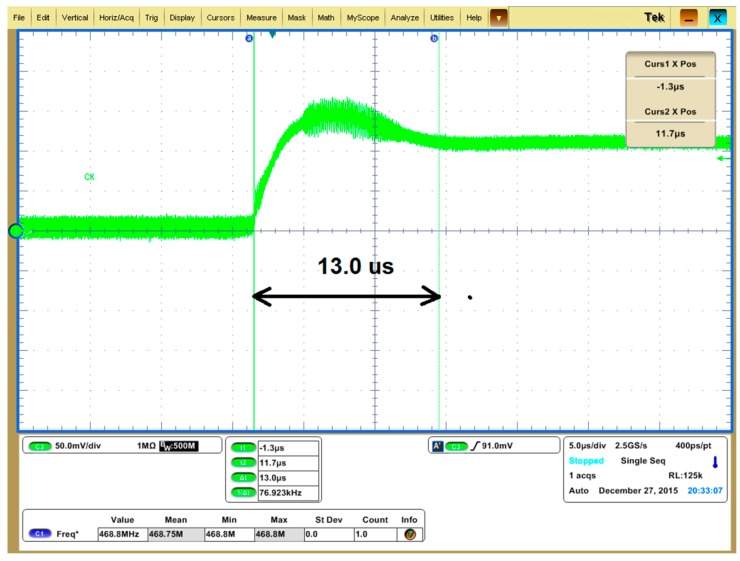
Measured PLL settling time. The curve shows the settling process of the VCO control voltage *V_C_* when the PLL frequency setting is changed.

The waveform of the PLL control voltage *V_C_* with a 1 V step is shown in Figure 21. The measured settling time is 13 μs, which is quite close to the simulation result. In this design, the searching step length ranges from 20 to 40 µs (4~8 IF clock cycles), which leaves enough margin for the PLL to settle down during the resonance frequency measurement.

**Figure 22:**
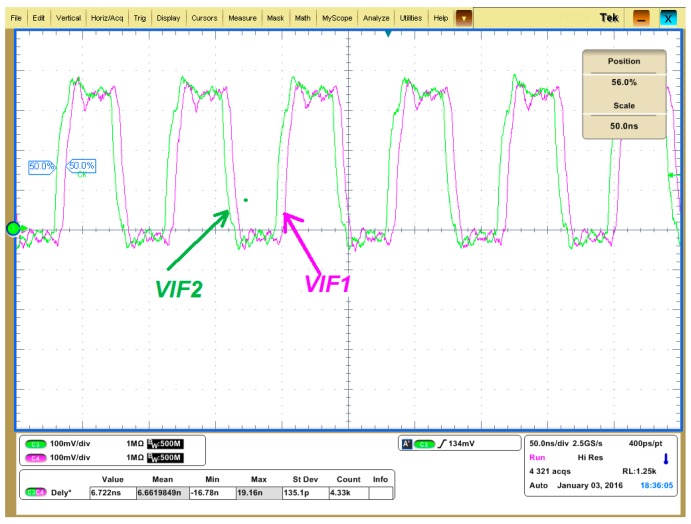
*V_IF1_* and *V_IF2_* when the PLL output frequency is less than the SAW resonance frequency.

**Figure 23:**
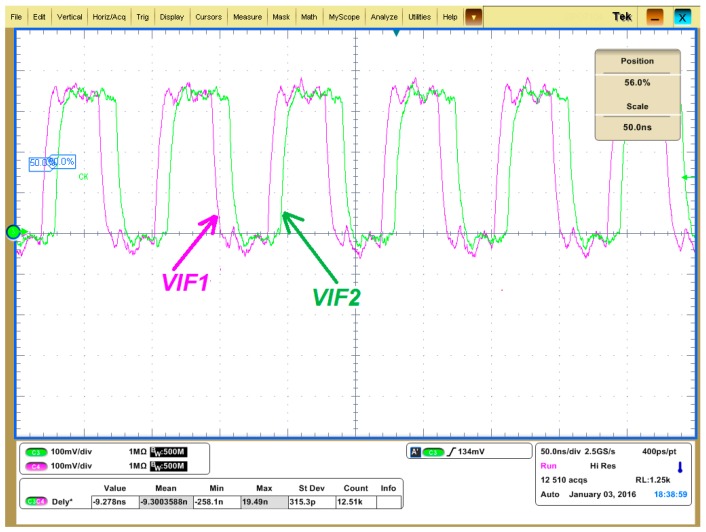
*V_IF1_* and *V_IF2_* when the PLL output frequency is larger than the SAW resonance frequency.

The waveforms of *V_IF1_* and *V_IF2_* (actually the single ended output of the comparators) are shown in Figure 22 and Figure 23. The green curve is *V_IF2_* which is the down converted signal of the SAW device driving amplifier output *V_RF2_*, and the pink line is *V_IF1_* which is the down converted signal of the SAW device driving amplifier input *V_RF1_*. The IF frequency is set to 12 MHz in Figure 22 and Figure 23 instead of 200 kHz to amplify the time difference. As shown in Figure 22, when the PLL frequency is less than the SAW resonance frequency *f_0_*, *V_IF2_* leads *V_IF1_*, which means *Δφ* is larger than 0, which agrees with Figure 4. Similarly, the case that *V_IF2_* lags *V_IF1_* as shown in Figure 23 means *Δφ* is less than 0, which indicates the PLL frequency is larger than the SAW resonance frequency *f_0_* according to Figure 4.

**Figure 24:**
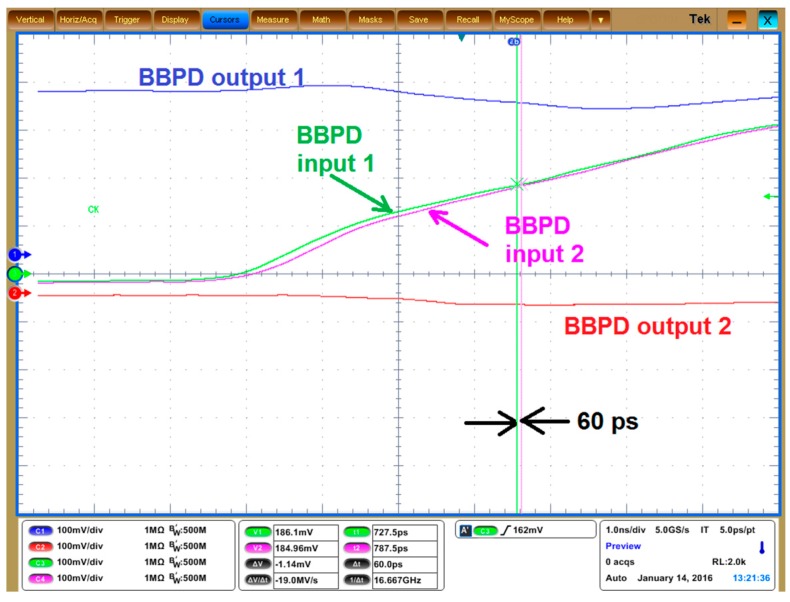
Measurement result of the minimum detectable time difference. When the time difference of the input signals to the BBPD is reduced down to 60 ps, the BBPD can still gives correct output.

The minimum detectable time difference of the BBPD is show in Figure 24. The green line and the pink lines are the BBPD input signals (the IF signals) to be differentiated, while the blue line and the red line are the differential outputs of the BBPD which tells the lead-lag relationship between the two input signals. It can be seen that the BBPD can give the correct output when the input difference is as small as 60 ps.

According to (7), (8) and (10), the frequency resolution limit imposed by the BBPD is calculated as

(25) $$\Delta f_{\min,BPFD}=\frac{\pi \cdot \Delta t_{PD}f_{IF}f_{0}}{Q}=262\;Hz$$

in which *Δt_PD_* = 60 ps as shown in Figure 24, *f_IF_* = 200 kHz, *f_0_* = 900MHz, *Q* =130. This number is smaller than the fractional-N PLL frequency resolution (773 Hz). Therefore the readout circuit frequency resolution is mainly constrained by the PLL frequency resolution.

**Figure 25:**
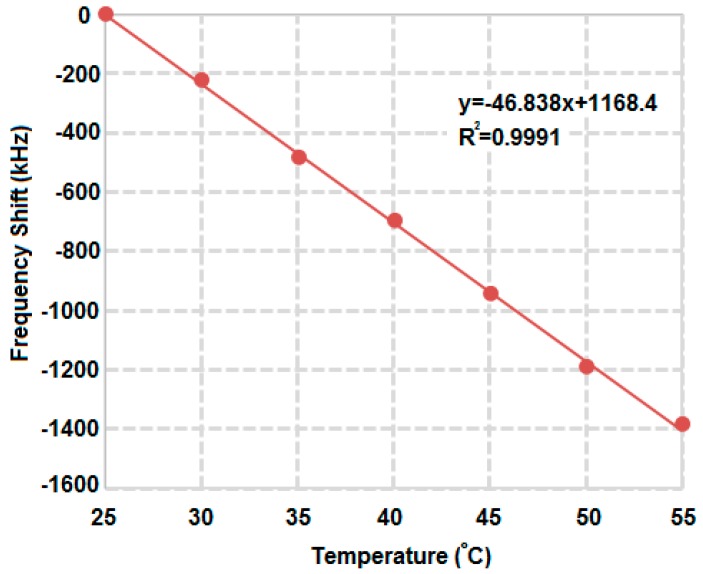
Measured SAW device resonance frequency shift against the environment temperature using the presented readout circuit.

The functionality of the proposed resonance readout circuit has been validated by using the readout chip and the SAW device as a temperature sensor. Figure 25 gives the measured SAW device resonance frequency shift versus the environment temperature using the measurement setup in Figure 18. The resonance frequency is calculated out of the PLL division ratio which represents the PLL frequency. The measurement sensitivity (the SAW device plus the readout circuit) is about −47 kHz/K with the linearity correlation coefficient *R*^2^ equal to 0.9991. This test validates the functionality of the presented SAW resonance frequency readout circuit.

Table 1 summarizes the performance of the presented resonance frequency readout circuit, and also gives the comparison between this work and the state-of-the-art results in literature. The measured power consumption of the SAW resonance frequency readout circuit is about 7 mW from a 1.6 V power supply. The frequency resolution is 733 Hz, and the relative measurement resolution, defined as the frequency resolution divided by the device resonance frequency, is 0.82 ppm, which is among the state-of-the-art results. It takes 0.48 ms for the readout circuit to determine the resonance frequency of the SAW device, which outperforms all the other work in literature. IOverall, this work has achieved a good trade-off between the relative accuracy and the measurement time performance.

## 5. Conclusions

A resonance frequency readout method is proposed for a 900 MHz SAW device in this paper. The proposed method is based on phase difference detection and SAR. It provides a good trade-off between the frequency measurement resolution and the measurement time. The readout circuit has been designed and fabricated in a 180 nm CMOS technology. The experimental results show that the proposed readout circuit has greatly improved the frequency measurement resolution, while the time required for a single measurement is shorter than the state-of-the-art results in literature. The functionality of the readout circuit has been tested with a 900 MHz RF SAW device as a temperature sensor. The presented readout circuit will be tested with the resonance-based pressure sensors and mass sensors for more applications in the future.

## Figures and Tables

**Table 1:** Performance summary and comparison.

Reference	This Work	ISSCC ‘12 [14]	TBioCAS 2012 [15]	Sensors 2014 [17]	JSSC‘16 [18]	JSSC’12 [20]	TMTT‘13 [19]	ISSCC ‘16 [34]
CMOS technology (nm)	180	250	250	350	350	90	90	180
Architecture	Phase-based	Time-based	Time-based	Time-based	Time-based	Freq.-based	Freq.-based	Freq. ratio
Q factor	130	280	450	386	376	N/A	N/A	N/A
Resonant frequency (Hz)	898 M	1.98 M	2.17 M	535.8 k	592 k	7~9 G	10.4 G	45 M
Frequency resolution (Hz)	733	53	5	26.8	17.6	2 M	156 k	N/A
Relative freq. resolution ^1^ (ppm)	0.82	26.77	2.3	50	29.8	>222	15	0.00028
Power cons. (mW)	7	1.35	1.35	0.1	0.06	16.5	22	19
Measurement time (ms)	0.48	5000	10,000	6	1.1	0.9	25	3.85

^1^ The relative frequency resolution is defined as the frequency resolution divided by the device resonance frequency.
